# Defining fallopian tube‐derived miRNA cancer signatures

**DOI:** 10.1002/cam4.2416

**Published:** 2019-09-10

**Authors:** Selam B. Dejene, Anders W. Ohman, Wei Du, Deepinder Randhawa, Anand Bradley, Niraj Yadav, Kevin M. Elias, Daniela M. Dinulescu, Sunita R. Setlur

**Affiliations:** ^1^ Department of Pathology Brigham and Women's Hospital Harvard Medical School Boston Massachusetts; ^2^ Division of Gynecologic Oncology, Department of Obstetrics, Gynecology, and Reproductive Biology Brigham and Women's Hospital, Dana‐Farber Cancer Institute, Harvard Medical School Boston Massachusetts

**Keywords:** BRCA, fallopian tube, microRNA, ovarian cancer, PTEN, TP53

## Abstract

**Background:**

MicroRNAs have recently emerged as promising circulating biomarkers in diverse cancer types, including ovarian cancer. We utilized conditional, doxycycline‐induced fallopian tube (FT)‐derived cancer models to identify changes in miRNA expression in tumors and plasma, and further validated the murine findings in high‐grade ovarian cancer patient samples.

**Methods:**

We analyzed 566 biologically informative miRNAs in doxycycline‐induced FT and metastatic tumors as well as plasma samples derived from murine models bearing inactivation of *Brca*, *Tp53*, and *Pten* genes. We identified miRNAs that showed a consistent pattern of dysregulated expression and validated our results in human patient serum samples.

**Results:**

We identified six miRNAs that were significantly dysregulated in doxycycline‐induced FTs (*P* < .05) and 130 miRNAs differentially regulated in metastases compared to normal fallopian tissues (*P* < .05). Furthermore, we validated miR‐21a‐5p, miR‐146a‐5p, and miR‐126a‐3p as dysregulated in both murine doxycycline‐induced FT and metastatic tumors, as well as in murine plasma and patient serum samples.

**Conclusions:**

In summary, we identified changes in miRNA expression that potentially accompany tumor development in murine models driven by commonly found genetic alterations in cancer patients. Further studies are required to test both the function of these miRNAs in driving the disease and their utility as potential biomarkers for diagnosis and/or disease progression.

## Graphical abstract

We have identified miRNAs that accompany fallopian tube‐derived ovarian cancer progression in murine models. Furthermore, we validated some of the miRNAs in patient serum samples.

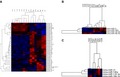

## 1 INTRODUCTION

Ovarian cancer is the fifth leading cause of cancer death among women and remains the leading cause of mortality among all gynecological cancers in the developed world. The poor survival rate primarily stems from the fact that it is a largely asymptomatic disease with 85% of cancers being diagnosed at advanced stages. The 5‐year survival rate increases from 29% to 80% if the cancer is diagnosed early.^1^ However, early diagnosis remains elusive due to the lack of effective biomarkers for asymptomatic patients with low volume, early stage disease. In addition, recent studies have proposed that a significant proportion of high‐grade serous cancers (HGSC), the most common and deadliest ovarian cancer subtype, may arise from the distal fallopian tubal epithelium (FTE)^2^, ^3^, ^4^ rather than the ovarian surface epithelium (OSE). These new findings have led investigators to search for molecular changes within dysregulated FTE that may accompany tumor development in patients and animal models, including FTE‐derived genetically engineered mouse models (GEMMs) targeting three key HGSC genetic alterations as shown by The Cancer Genome Atlas (TCGA) and previous studies^5^: *Brca1/2(+/- or ‐/‐); Tp53(R172H/‐);* and *Pten(‐/‐)* via a conditional Pax8‐Cre doxycycline‐inducible system.^6^

The quest for noninvasive biomarkers has recently expanded to include microRNAs (miRNA), a class of small noncoding RNAs (18‐22 nt) that regulate 30% of protein coding genes.^7^ The mechanism by which miRNA regulate gene expression posttranscriptionally is through their binding to 3' untranslated regions (UTR) of target mRNAs further leading to mRNA decay and/or translational repression.^8^ It is worth noting that miRNAs are attractive as circulating biomarkers because they are inherently stable in serum and plasma. Interestingly, multiple groups have already identified several circulating miRNAs of interest in ovarian cancer.^9^, ^10^, ^11^, ^12^ Some of these studies show a correlation between miRNA expression patterns in tissues and plasma/serum. In addition, a recent investigation has employed a complex neural network analysis to identify miRNA signatures from samples obtained prior to surgery and chemotherapy.^12^ The same report showed that the miRNA model performed better than the CA‐125 biomarker in being able to better diagnose ovarian cancer. All these studies have utilized serum from patients with ovarian cancer, most of whom have been diagnosed at advanced stages. There is still no clear understanding of the changes in miRNA expression that may occur during pathogenesis in the FTE nor the relationship between miRNA expression in the FTE and metastatic tumors. In this study, we sought to delineate miRNA changes following doxycycline induction and compared these changes to miRNA patterns seen in patient samples with and without disease.

## 2 MATERIALS AND METHODS

### 2.1 RNA extraction

The samples used for the study are summarized in the Table S1. These included doxycycline‐induced fallopian tube samples (FT, n = 5), metastatic tumors (M, n = 6), and uninduced control fallopian tubes (CT, n = 5) as well as plasma samples from mice bearing tumors (TS, n = 4) and normal control mice (CS, n = 4). Total RNA was isolated from tissue and plasma samples using the Norgen's Animal Tissue RNA Purification Kit and Plasma/Serum RNA Purification Kits, respectively, (Norgen Biotek Corporation) as per manufacturer's instructions. The isolated RNA was quantified using the Qubit™ RNA HS Assay Kit (Life Technologies, Thermo Fisher Scientific Inc).

### 2.2 Nanostring analysis

miRNAs from all samples (shown in the Table S1) were profiled using the nCounter® miRNA Expression Assays v1.5 (NanoString Technologies). The NanoString data were analyzed using the nSolver 3.0 software with their recommended settings (Figure S1). Briefly, tissue samples were normalized to the geometric mean of top 100 miRNAs per recommended settings without any background subtraction. Since plasma is known to have low abundance of miRNAs compared to tissues, we first determined the total number of miRNAs that were expressed (expression above the average of negative ligation controls plus two standard deviations). We found approximately 150 miRNAs that were expressed. Since nSolver recommends using top 100 miRNAs for normalization, which represents approximately the top 20% of miRNAs expressed in tissues, we normalized plasma sample data to the geometric mean of top 30 miRNAs. The metastatic tumor sample M5 failed the normalization QC and was excluded from further analysis. Similarly, the control plasma sample CS1 was excluded from the analysis. Therefore, the final number of samples analyzed included CT (n = 5), FT (n = 5), M (n = 5), CS (n = 3), and TS (n = 4). The statistical difference in miRNA expression between control and tumor samples was calculated using a two‐tailed t test (Tables S2‐S4). The resulting list of differentially expressed miRNAs was analyzed for homology to human miRNA (microRNAviewer, Broad Institute of Harvard and MIT^13^; miRbase (release 22^14^).

### 2.3 Quantitative PCR (qPCR) analysis

miRNA expression was validated using the Quanta Biosciences qScript assay. Primer sequences for qPCR, which were predesigned by Quanta Biosciences, were ordered from IDT (Table S5). The samples used for qPCR were the same samples employed for the NanoString discovery analysis. We utilized miR‐425 as the endogenous control owing to its observed stability in the NanoString dataset (lowest % coefficient of variation across samples, comparable to the positive controls). hsa‐miR‐425, has also previously been reported to be one of the most stably expressed endogenous (EC) miRNA.^15^ Five hundred nanograms of RNA from each sample was converted to cDNA using the cDNA Synthesis Kit (Quanta BioSciences). All cDNA samples were run in triplicate using the PerfeCTa SYBR Green SuperMix ROX (Quanta BioSciences). The qPCR was performed on an Applied Biosystems 7300 Real‐Time PCR System using a two‐step cycling protocol (95°C for 2 minutes followed by 40 cycles of 95°C for 15s and 62°C for 30 seconds, except for miR‐34b‐3p for which the annealing temperature was 60°C for 60 seconds). miRNA expression was quantified using the comparative Ct method.

### 2.4 Validation in human patient samples

Selected miRNA‐seq data in tags per million (tpm) were downloaded from the Gene Expression Omnibus (GEO) using a publicly available dataset (GSE94533) of preoperative serum samples collected from both healthy controls and women with malignant adnexal masses.^12^ Data were filtered for miRNAs with a minimum threshold of 10 tpm, log‐transformed, and expressed as fold changes relative to an established set of nine reference serum miRNAs described in our prior publication: let‐7c‐5p, miR‐103a‐3p, miR‐146b‐5p, miR‐148b‐3p, miR‐181a‐5p, miR‐191‐5p, miR‐221‐3p, miR‐222‐3p, and miR‐423‐3p.^12^ Samples were classified as either healthy controls or cancer cases using histopathologic diagnosis and the International Federation of Obstetrics and Gynecology (FIGO) stage guidelines. Cases were grouped into stage I serous borderline tumors, stage I or II high‐grade (Grade 3) serous cancers, stage I or II high‐grade (Grade 2 or 3) clear cell or endometrioid cancers, stage III or IV high‐grade (Grade 3) serous cancers, stage III or IV high‐grade (Grade 2 or 3) clear cell or endometrioid cancers. Mean expression values of miRNAs of interest were compared among groups using a one‐way analysis of variance (ANOVA) analysis with a posttest for linear trend to test the null hypothesis of no variation in values with increasing burden of disease. An alpha statistic less than 0.05 was considered statistically significant. Data were analyzed using the GraphPad Prism v.8.0 software (San Diego, CA).

## 3 RESULTS

**Figure 1:**
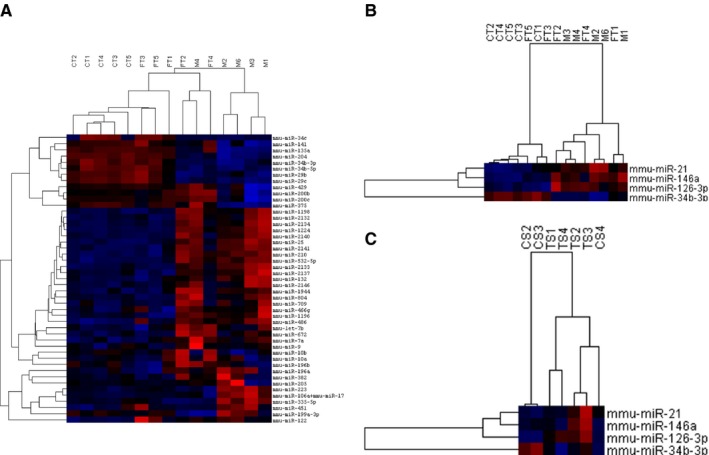
Hierarchical clustering. Unsupervised agglomerative clustering analysis of tissue samples (A). Supervised clustering analysis with miR‐21a‐5p, miR‐126a‐3p, miR‐146a‐5p, and miR‐34b‐3p in tissue (B) and plasma (C). In the heatmaps, blue denotes underexpression and red denotes overexpression of the miRNAs

We performed a miRNA profiling analysis in order to identify changes occurring in doxycycline‐induced FTs and metastatic tumors associated with inactivation of *BRCA*, *TP53*, and *PTEN* genes, which may accompany tumorigenesis and may be of use as potential circulating biomarkers. Accordingly, total RNA was isolated from selected doxycycline‐induced FT, metastatic tumors, and plasma samples. We had matching doxycycline‐induced FTs and metastatic tumors from two mice. We included three other metastatic tumors from a previous publication.^6^ Normal (uninduced) FTs were used as controls. We also used plasma samples from all the mice from which we collected doxycycline‐induced FT tissue. The miRNA expression was profiled using the NanoString platform and the workflow is summarized in the Figure S1. Unsupervised agglomerative hierarchical clustering of the normalized data using Pearson's correlation shows separation of the tissue samples into two separate clusters representing normal control FT (CT), the doxycycline‐induced FT, and metastatic tumors (M) (Figure 1A). We identified miRNAs that were differentially expressed in doxycycline‐induced FTs and metastatic tumor samples (M) when compared to CT (Tables S2, S3). The miRNAs that showed significant differential expression (*P* value ≤.05, two‐tailed t test) in doxycycline‐induced FTs are shown in Table 1. In addition, we identified 130 murine miRNAs that were significantly differentially expressed in metastatic tumor samples, M, when compared to uninduced FT controls (*P* value <.05, two‐tailed t test). We then examined all the differentially expressed miRNAs in doxycycline‐induced FT and M, when compared to the controls, to identify miRNAs that were consistently upregulated in both lists. We further selected the top 50 overexpressed miRNAs in doxycycline‐induced FT, which were also significantly overexpressed in metastatic tumors. The list of top 50 overexpressed murine miRNAs was subjected to homology analysis (microRNAviewer, Broad Institute of Harvard and MIT^13^) to identify human homologs, which led to the identification of 15 miRNAs (Table 2). miR‐2134, miR‐465b‐5p, miR‐2141, miR‐1970, miR‐2132, miR‐672‐5p, miR‐1944, miR‐292a‐3p, miR‐1198‐5p, miR‐291b‐3p, and miR‐2140 did not have human homologs and were therefore excluded from the list. Evaluation of these miRNAs in plasma showed that all the selected miRNAs had a twofold change in expression, except for miR‐126a‐3p, which had an expression fold change of 1.88 as shown in Table 3 and Table S4. Of the selected miRNAs, miR‐210‐3p and miR‐486a‐5p were not studied further as their levels were reported to be highly impacted by hemolysis and would therefore not be suitable as potential circulating tumor markers.^16^ Interestingly, the supervised clustering analysis using the consistently dysregulated miRNAs, including miR‐21a‐5p, miR‐126a‐3p, miR‐146a‐5p, and miR‐34b‐3p, shows a good separation between normal control and doxycycline‐induced samples (Figure 1B,C).

**Table 1:** Significant differentially expressed miRNAs in doxycycline‐induced FT

Gene name	Accession Number	Fold change: fallopian tube vs control	*P*‐value: fallopian tube vs control	Fold change M vs CT	*P*‐value M vs CT
mmu‐miR‐486‐5p	MIMAT0003130	4.05	2.00E‐02	8.37	1.00E‐03
mmu‐miR‐21a‐5p	MIMAT0000530	1.92	3.00E‐02	4.59	2.00E‐03
mmu‐miR‐495‐3p	MIMAT0003456	1.65	4.00E‐02	1.58	2.50E‐01
mmu‐miR‐432‐5p	MIMAT0012771	−1.83	4.00E‐02	−1.29	3.30E‐01
mmu‐miR‐146a‐5p	MIMAT0000158	2.28	5.00E‐02	4.43	1.00E‐03
mmu‐miR‐7a‐5p	MIMAT0000677	2.15	5.00E‐02	4.07	3.00E‐02
mmu‐miR‐34b‐3p	MIMAT0004581	−3.98	6.00E‐02	−16.97	2.00E‐04

**Table 2:** Significantly upregulated miRNAs in M samples that are also upregulated in doxycycline‐induced FT

miRNA	Fold change M vs CT	*P‐*value M vs CT	Fold Change FT vs CT	*P*‐value FT vs CT
mmu‐miR‐22‐3p	4.92	5.08E‐06	2.08	2.15E‐01
mmu‐miR‐210‐3p	12.13	5.78E‐05	3.01	1.59E‐01
mmu‐miR‐532‐5p	10.48	8.74E‐05	2.91	1.41E‐01
mmu‐let‐7i‐5p	5.8	3.00E‐04	2.98	1.56E‐01
mmu‐miR‐146a‐5p	4.43	7.00E‐04	2.28	4.61E‐02
mmu‐miR‐486a‐5p	8.37	1.00E‐03	4.05	1.90E‐02
mmu‐miR‐126a‐3p	3.07	1.00E‐03	2.25	2.12E‐01
mmu‐miR‐21a‐5p	4.59	1.50E‐03	1.92	2.92E‐02
mmu‐miR‐1224‐5p	41.91	3.00E‐03	3	2.18E‐01
mmu‐miR‐122‐5p	5.04	4.60E‐03	7.47	6.01E‐02
mmu‐miR‐98‐5p	3.44	1.24E‐02	2.29	1.40E‐01
mmu‐miR‐450b‐5p	3.05	2.05E‐02	2.25	1.70E‐01
mmu‐miR‐363‐3p	3.4	2.86E‐02	2.46	7.51E‐02
mmu‐miR‐7a‐5p	4.07	3.02E‐02	2.15	4.85E‐02
mmu‐miR‐10b‐5p	–1.95	4.41E‐02	1.86	2.15E‐01

**Table 3:** Fold change of consistently upregulated miRNAs in plasma and tissue samples

miRNA	Fold change in plasma	Fold change in FT	Fold change in M
mmu‐miR‐126a‐3p	1.88	2.25	3.07
mmu‐miR‐1224‐5p	2.29	3	41.91
mmu‐miR‐146a‐5p	2.34	2.28	4.43
mmu‐miR‐21a‐5p	2.56	1.92	4.59

### 3.1 Validation of miRNA expression

**Figure 2:**
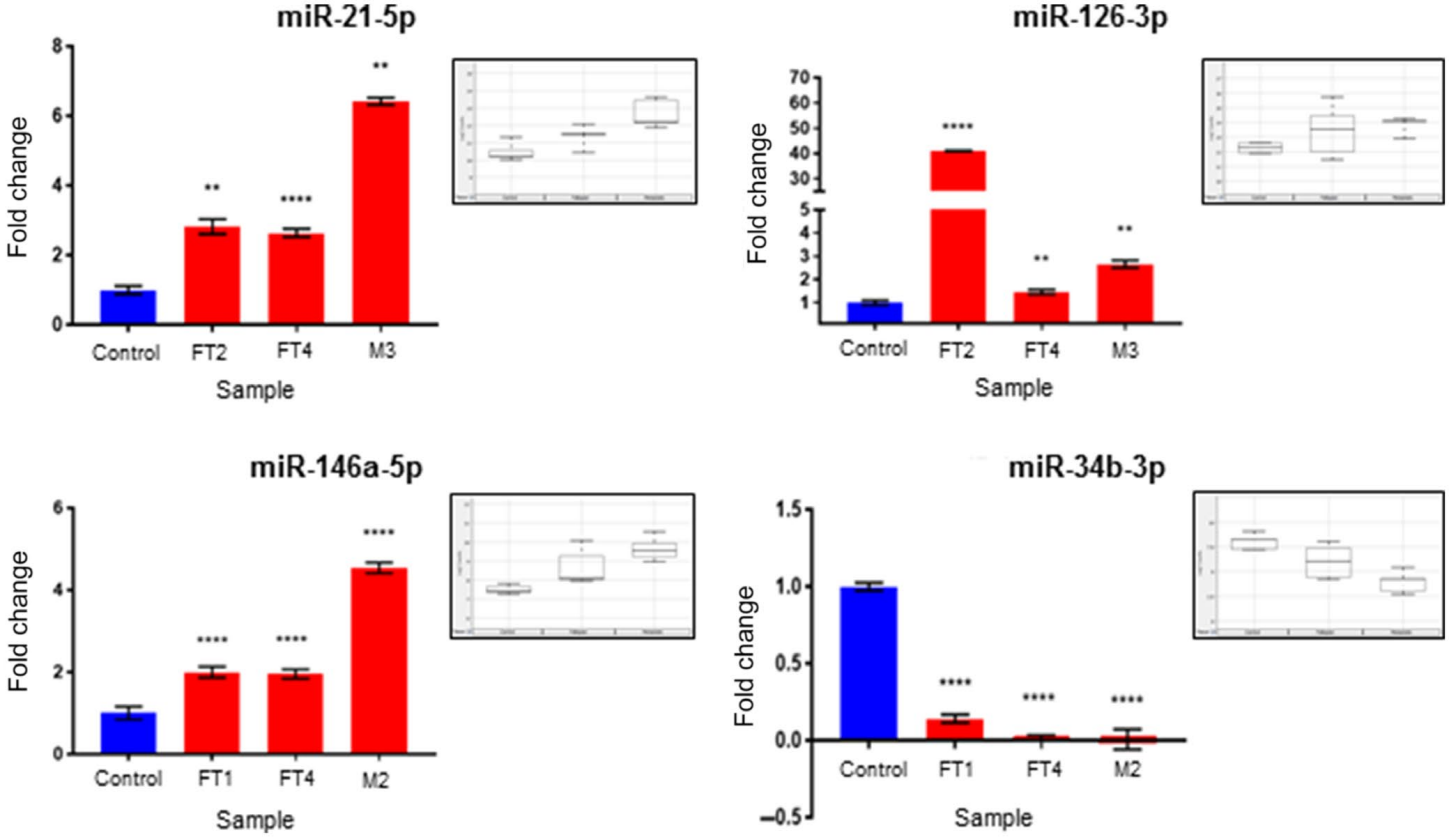
qPCR validation studies showing expression fold changes of miR‐21, miR‐126‐3p, miR‐146a, and miR‐34b‐3p in doxycycline‐induced fallopian tubes (FT2, FT4) and metastatic samples (M2 and M3) when compared to Controls. Statistical significance was calculated by using an unpaired t test with Welch's correction (**** and ** indicate *P* <.0001 and <.005, respectively). The box plots in the insets show miR‐21a‐5p, miR‐126a‐3p, miR‐146a‐5p, and miR‐34b‐3p expression in tissue samples as determined by the NanoString analysis

**Figure 3:**
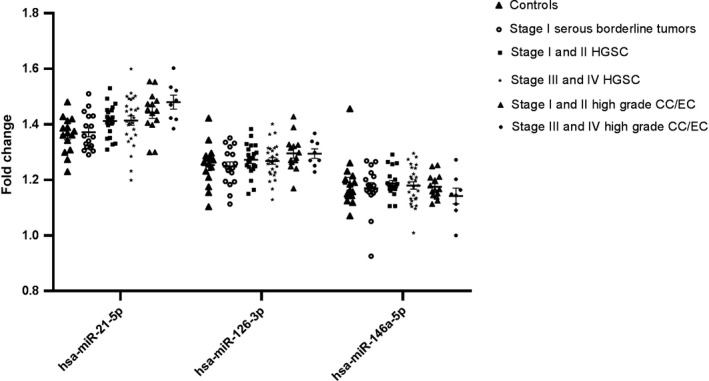
Validation of selected murine miRNAs of interest in patient samples. Compared to healthy controls and borderline serous tumors, increasing stage of both high‐grade serous and non‐serous ovarian cancers was associated with a progressive rise in miR‐21‐5p (*P*‐trend <.0001) and miR‐126‐3p (*P*‐trend = .03). Higher volume disease was not associated with levels of miR‐146a‐5p (*P‐*trend = .2), which was due primarily to decreased expression among advanced‐stage non‐serous cancers. Data are shown as mean values ± standard error of the mean (SEM)

We performed qPCR analysis using the same samples in order to validate the results obtained with the NanoString analysis. Of all the miRNAs identified by the expression profiling analysis, only those that showed consistent expression patterns in doxycycline‐induced tissue (FT, M) and plasma samples were further selected for validation. Thus, we validated by qPCR the following miRNAs: miR‐21a‐5p, miR‐126a‐3p, miR‐146a‐5p, and miR‐34b‐3p (Figure 2). Finally, to test if the identified murine miRNAs have clinical relevance in human samples, we further performed a validation analysis utilizing a recently published patient dataset, which examined circulating miRNAs in human patients diagnosed with ovarian cancer.^12^ The relevant clinical characteristics of the cancer patients are described in Table 4. miR‐34b‐3p was not detectable in any of the human serum samples above the minimum threshold for detection of 10 tpm. It has, however, been shown to be downregulated in human serous ovarian cancer.^17^ Interestingly, two of the key miRNAs we identified in animal models, namely miR‐21a‐5p and miR‐126‐3p, showed a trend for increasing serum miRNA levels with increased tumor stage in HGSC as well as in non‐serous histology (Figure 3). Specifically, compared to healthy controls and borderline serous tumors, we saw increasing expression of both miR‐21‐5p (*P*‐trend <.0001) and miR‐126‐3p (*P*‐trend = .03) with increased stage of both high‐grade serous and non‐serous ovarian cancers. miR‐146a‐5p levels were not found to be significantly correlated with progressive disease in patients, which was due primarily to decreased expression among advanced‐stage non‐serous cancers. However, this miRNA has been previously shown to be upregulated in serous ovarian cancer (Figure 3).^18^

**Table 4:** Clinical characteristics of patient samples

	N	Age, y	CA‐125, IU/L
Healthy Controls	15	53 (45‐71)	**
Stage I Serous Borderline tumors	17	56 (45‐69)	42 (4.9‐763)
Stage I/II High‐Grade Serous Carcinomas	21	59 (45‐74)	174 (9‐7000)
Stage I/II High‐Grade Endometrioid or Clear Cell Carcinomas	14	59 (48‐78)	300 (25.8‐7005)
Stage III/IV High‐Grade Serous Carcinomas	27	56 (46‐68)	157.3 (31.2‐1889)
Stage III/IV High‐Grade Endometrioid or Clear Cell Carcinomas	8	55.5 (50‐63)	551.65 (58‐3257)

Note

Values represent medians (range).

** Not applicable.

## 4 DISCUSSION

The identification of the FT as a site of origin for a large subset of HGSC necessitates a detailed investigation into the early changes that accompany tubal‐derived tumorigenesis. In order to characterize these early changes and discover potential circulating biomarkers, we utilized FT‐derived cancer models and identified miRNAs that are expressed early in doxycycline‐induced FTs, retain consistent expression patterns in metastatic tumors, and can be detected as circulating miRNAs in plasma/serum samples. Furthermore, we have shown that selected murine miRNAs appear to be reflective of disease progression in human samples as well. This could be useful for disease management and patient selection for primary debulking surgery by differentiating patients with low‐volume from high‐volume disease. There was a trend for differential expression of miRNAs in our dataset between serous and non‐serous tumors, suggesting unique biomarker properties segregated by histology, although these differences did not reach statistical significance. This will be an area of future investigation with an expanded cohort.

Interestingly, some of the miRNAs identified in our study have also been previously reported as circulating biomarkers in ovarian cancer by other groups. These include miR‐126a‐3p^19^ and miR‐21a‐5p.^20^ These data not only validate the animal models in recapitulating the clinical disease but also point to a potential key role for circulating biomarkers during tumorigenesis. In addition, we found miRNAs which were regulated by the very genes targeted to initiate tumorigenesis in murine models, namely *Brca1/2*, *Tp53*, and *Pten*. One of these miRNAs is miR‐34b‐3p, a member of the miR‐34 family of tumor suppressor microRNA, which has been shown to be downregulated in ovarian surface epithelial cells following *TP53* inactivation.^17^ A recent integrated analysis of miRNA‐mRNA networks in familial ovarian cancer showed mir‐34b‐3p to be downregulated and at the center of the interaction network.^21^ The members of the miR‐34 family are activated by *TP53* in normal cells.^22^, ^23^ miR‐34b‐3p was found to be downregulated in our murine models, thus supporting the earlier report that this miRNA may mediate the downstream effects of *TP53* knockdown on cell proliferation and adhesion‐independent growth.^17^ Although we did not detect miR‐34b‐3p in human circulation in our small patient sample size, it has been reported to be downregulated in serous ovarian cancer.^17^ Similarly, miR‐21a‐5p, which is normally downregulated by *PTEN*,^21^ was found to be upregulated following conditional *Pten* inactivation in murine tumor models and further validated as discriminating between serous cancers and control cases in the patient analysis. miR‐21a‐5p has been reported previously as overexpressed in ovarian tumors by multiple studies^20^, ^24^ and is being tested for potential use as a circulating tumor biomarker for ovarian cancer detection.^25^ In addition, a recent study has found that miR‐21a‐5p plays a role in epithelial to mesenchymal transition, demonstrating its oncogenic potential.^21^ Therefore, the effects of *PTEN* knockdown in tumorigenesis may be also mediated by this miRNA. Finally, a third murine miRNA, which we identified as upregulated in murine models was miR‐146a‐5p that regulates *Brca1*.^26^ As we did not have *BRCA* mutation information in the patient cohort, it is possible that differences seen in miR‐146a‐5p expression patterns between mice and humans may be due to a low proportion of *BRCA* mutation carriers in our patient study. This particular miRNA has, however, been reported to be overexpressed in human serous ovarian cancers in another study.^18^ Interestingly, in breast cancer, miR‐146a has been reported as being overexpressed in tumors with *BRCA1/2* mutations.^26^ Furthermore, miR‐146a‐5p has also been shown to play a role in ovarian cancer progression and its expression is positively correlated with survival and resistance to platinum.^18^, ^27^ This miRNA was upregulated in our murine models that bear *Brca1/2* deletions. One possible mechanism by which miR‐146a‐5p upregulation may occur is via *PTEN* knockdown‐induced overexpression of FOXP3. FOXP3 has been shown to be responsible for miR‐146a overexpression.^28^, ^29^ In addition, FOXP3 is itself regulated by *PTEN* and is overexpressed following *PTEN* knockdown.^30^ miRNAs are estimated to regulate a large proportion of protein coding genes in cells and therefore have a significant impact on modulating cellular pathways.^25^ Hence, these miRNA pathways may be activated by the key genetic alterations found in patients and targeted in murine models and function in concert to potentially aid the tumorigenic process in tubal‐derived cancer models.

In summary, our analysis has provided insights into mapping potential functional pathways induced by *Brca*, *Tp53*, and *Pten* alterations in the induced murine FT and metastatic tumors. Furthermore, we have found that some of the identified miRNAs regulate or are regulated by key genetic tumorigenic drivers and if validated in patients, may have applicability as potential clinical biomarkers for diagnosis and/or disease progression.

## CONFLICT OF INTEREST

The authors confirm that there are no potential conflicts of interest to declare.

## Data Availability

The data has been uploaded to GEO (GSE126549) and is publicly available.
