# Supplementary material for: Defining fallopian tube‐derived miRNA cancer signatures
Source: Cancer Med. 2019 Sep 10;8(15):6709–16. doi: 10.1002/cam4.2416 (PMC6825987; doi:10.1002/cam4.2416)
Supplement: Supplementary file 2 [file CAM4-8-6709-s002.docx]

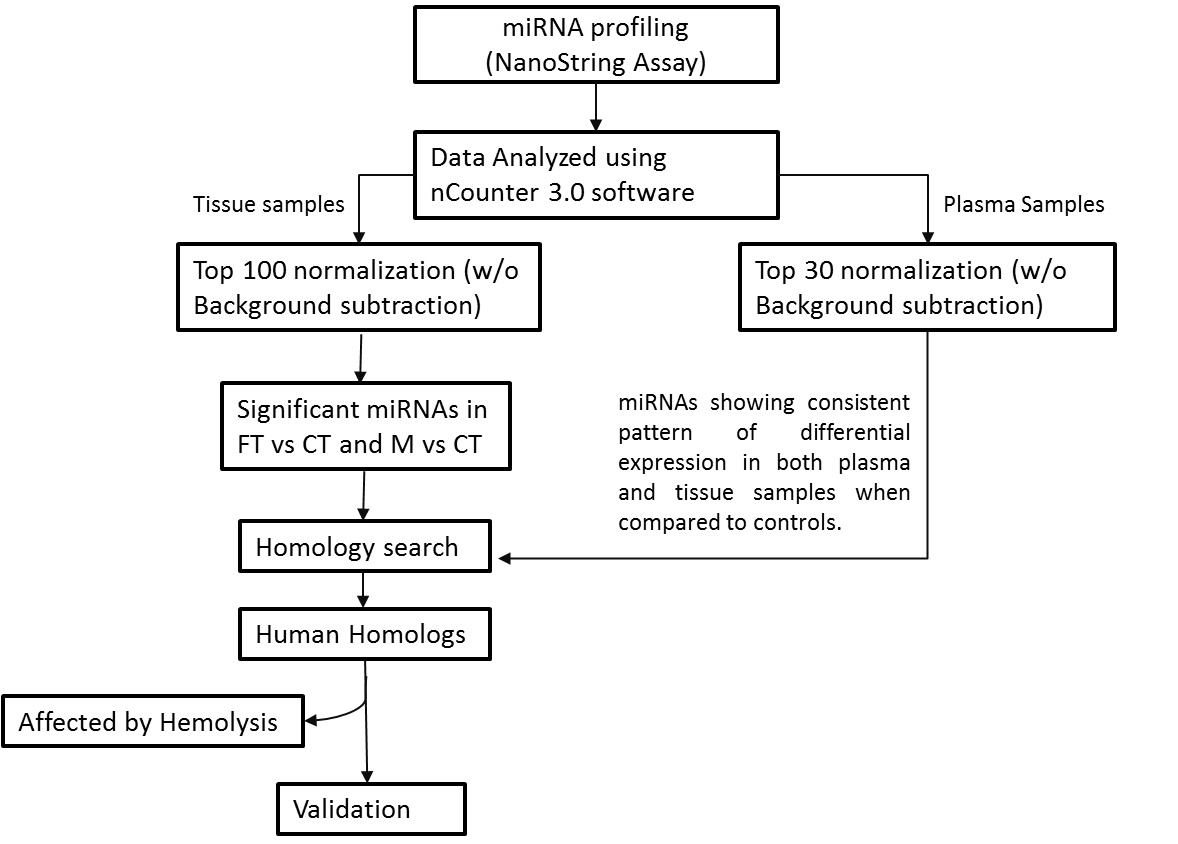


**Supplementary Figure S1.** Study workflow. The flowchart summarizes the workflow for NanoString profiling analysis

**Supplementary Table S1: Genotypes of tissue and plasma samples included in the study**

| Genotype, Control Sample | Tissue ID | Plasma sample ID |
| --- | --- | --- |
| *Brca1 (fl/fl); Tp53 (fl/fl); Pten (fl/fl)* | CT1 | CS1 |
| *Brca1 (fl/+); Tp53 (fl/fl); Pten (fl/fl)* | CT2 | CS2 |
| *Brca1 (fl/fl); Tp53 (LSL•R172H/fl); Pten (fl/fl)* | CT3 | CS3 |
| *Brca2 (fl/fl); Tp53 (fl/fl); Pten (fl/fl)* | CT4 | CS4 |
| *Brca2 (fl/+); Tp53 (LSL•R172H/fl); Pten (fl/fl)* | CT5 |  |
| Genotype, doxycycline-induced Fallopian Tube |  |  |
| *Brca2 (-/+); Tp53 (-/-); Pten (-/-)* | FT1 |  |
| *Brca1 (-/-); Tp53 (-/-); Pten (-/-)* | FT2 | TS1 |
| *Brca2 (-/+); Tp53 R172H/-); Pten (-/-)* | FT3 | TS2 |
| *Brca2 (-/-); Tp53 (-/-); Pten (-/-)* | FT4 | TS3 |
| *Brca2 (-/+); Tp53 (R172H/-); Pten (-/-)* | FT5 | TS4 |
| Genotype, Peritoneal Metastatic Tumor |  |  |
| *Brca2 (-/-); Tp53 (R172H/-); Pten (-/-)* | M1 |  |
| *Brca1 (-/+); Tp53 (-/-); Pten (-/-)* | M2 |  |
| *Brca2 (-/-); Tp53 (R172H/-); Pten (-/-)* | M3 |  |
| *Brca2 (-/+); Tp53 (-/-); Pten (-/-)* | M4 |  |
| *Brca2 (-/-); Tp53 (-/-); Pten (-/-)* | M5 |  |
| Genotype, Ovarian Metastatic Tumor |  |  |
| *Brca1 (-/+); Tp53 (-/-); Pten (-/-)* | M6 |  |

**Supplementary Table S5: Primer sequences of miRNAs used for qPCR**

| **miRNA** | **Forward Primer** | **Reverse Primer** |
| --- | --- | --- |
| mmu-miR-425 | ACACGATCACTCCCGTTGAA |  |
| mmu-miR-21 | GCTAGCTTATCAGACTGATGTTGAAA |  |
| mmu-miR-126-3p | CGTACCGTGAGTAATAATGCGAAA |  |
| mmu-miR-146a-5p | TGAGAACTGAATTCCATGGGTTA |  |
| mmu-miR-34b-3p | CAATCACTAACTCCACTGCCATAA |  |
| PerfeCTa® Universal PCR Primer |  | ATGGCGGTAAGTCCAGATACG |
